# Cumulative Probability of False-Positive Results After 10 Years of Screening With Digital Breast Tomosynthesis vs Digital Mammography

**DOI:** 10.1001/jamanetworkopen.2022.2440

**Published:** 2022-03-25

**Authors:** Thao-Quyen H. Ho, Michael C. S. Bissell, Karla Kerlikowske, Rebecca A. Hubbard, Brian L. Sprague, Christoph I. Lee, Jeffrey A. Tice, Anna N. A. Tosteson, Diana L. Miglioretti

**Affiliations:** 1 Division of Biostatistics, Department of Public Health Sciences, University of California Davis School of Medicine, Davis; 2 Department of Training and Scientific Research, University Medical Center, Ho Chi Minh City, Vietnam; 3 General Internal Medicine Section, Department of Veterans Affairs, University of California, San Francisco; 4 Department of Medicine, University of California, San Francisco; 5 Department of Epidemiology and Biostatistics, University of California, San Francisco; 6 Department of Biostatistics, Epidemiology & Informatics, University of Pennsylvania Perelman School of Medicine, Philadelphia, Pennsylvania; 7 Department of Surgery, Office of Health Promotion Research, Larner College of Medicine at the University of Vermont and University of Vermont Cancer Center, Burlington, Vermont; 8 Department of Radiology, University of Washington School of Medicine, Seattle; 9 Department of Health Systems and Population Health, University of Washington School of Public Health, Seattle; 10 Hutchinson Institute for Cancer Outcomes Research, Seattle, Washington; 11 Division of General Internal Medicine, Department of Medicine, University of California, San Francisco; 12 The Dartmouth Institute for Health Policy and Clinical Practice, Dartmouth College, Lebanon, New Hampshire; 13 Department of Medicine, Geisel School of Medicine at Dartmouth, Hanover, Lebanon, New Hampshire; 14 Department of Oncology, Norris Cotton Cancer Center, Lebanon, New Hampshire; 15 Kaiser Permanente Washington Health Research Institute, Kaiser Permanente Washington, Seattle

## Abstract

**Importance:**

Breast cancer screening with digital breast tomosynthesis may decrease false-positive results compared with digital mammography.

**Objective:**

To estimate the probability of receiving at least 1 false-positive result after 10 years of screening with digital breast tomosynthesis vs digital mammography in the US.

**Design, Setting, and Participants:**

An observational comparative effectiveness study with data collected prospectively for screening examinations was performed between January 1, 2005, and December 31, 2018, at 126 radiology facilities in the Breast Cancer Surveillance Consortium. Analysis included 903 495 individuals aged 40 to 79 years. Data analysis was conducted from February 9 to September 7, 2021.

**Exposures:**

Screening modality, screening interval, age, and Breast Imaging Reporting and Data System breast density.

**Main Outcomes and Measures:**

Cumulative risk of at least 1 false-positive recall for further imaging, short-interval follow-up recommendation, and biopsy recommendation after 10 years of annual or biennial screening with digital breast tomosynthesis vs digital mammography, accounting for competing risks of breast cancer diagnosis and death.

**Results:**

In this study of 903 495 women, 2 969 055 nonbaseline screening examinations were performed with interpretation by 699 radiologists. Mean (SD) age of the women at the time of the screening examinations was 57.6 (9.9) years, and 58% of the examinations were in individuals younger than 60 years and 46% were performed in women with dense breasts. A total of 15% of examinations used tomosynthesis. For annual screening, the 10-year cumulative probability of at least 1 false-positive result was significantly lower with tomosynthesis vs digital mammography for all outcomes: 49.6% vs 56.3% (difference, –6.7; 95% CI, –7.4 to –6.1) for recall, 16.6% vs 17.8% (difference, –1.1; 95% CI, –1.7 to –0.6) for short-interval follow-up recommendation, and 11.2% vs 11.7% (difference, –0.5; 95% CI, –1.0 to –0.1) for biopsy recommendation. For biennial screening, the cumulative probability of a false-positive recall was significantly lower for tomosynthesis vs digital mammography (35.7% vs 38.1%; difference, –2.4; 95% CI, –3.4 to –1.5), but cumulative probabilities did not differ significantly by modality for short-interval follow-up recommendation (10.3% vs 10.5%; difference, –0.1; 95% CI, –0.7 to 0.5) or biopsy recommendation (6.6% vs 6.7%; difference, –0.1; 95% CI, –0.5 to 0.4). Decreases in cumulative probabilities of false-positive results with tomosynthesis vs digital mammography were largest for annual screening in women with nondense breasts (differences for recall, –6.5 to –12.8; short-interval follow-up, 0.1 to –5.2; and biopsy recommendation, –0.5 to –3.1). Regardless of modality, cumulative probabilities of false-positive results were substantially lower for biennial vs annual screening (overall recall, 35.7 to 38.1 vs 49.6 to 56.3; short-interval follow-up, 10.3 to 10.5 vs 16.6 to 17.8; and biopsy recommendation, 6.6 to 6.7 vs 11.2 to 11.7); older vs younger age groups (eg, among annual screening in women ages 70-79 vs 40-49, recall, 39.8 to 47.0 vs 60.8 to 68.0; short-interval follow-up, 13.3 to 14.2 vs 20.7 to 20.9; and biopsy recommendation, 9.1 to 9.3 vs 13.2 to 13.4); and women with entirely fatty vs extremely dense breasts (eg, among annual screening in women aged 50-59 years, recall, 29.1 to 36.3 vs 58.8 to 60.4; short-interval follow-up, 8.9 to 11.6 vs 19.5 to 19.8; and biopsy recommendation, 4.9 to 8.0 vs 15.1 to 15.3).

**Conclusions and Relevance:**

In this comparative effectiveness study, 10-year cumulative probabilities of false-positive results were lower on digital breast tomosynthesis vs digital mammography. Biennial screening interval, older age, and nondense breasts were associated with larger reductions in false-positive probabilities than screening modality.

## Key Points

**Question:**

Is there a difference between screening with digital breast tomosynthesis vs digital mammography in the probability of false-positive results after 10 years of screening?

**Findings:**

In this comparative effectiveness study of 903 495 individuals undergoing 2 969 055 screening examinations, the 10-year cumulative probability of receiving at least 1 false-positive recall was 6.7% lower for tomosynthesis vs digital mammography with annual screening and 2.4% lower for tomosynthesis vs digital mammography with biennial screening, a significant difference.

**Meaning:**

The findings of this study suggest that digital breast tomosynthesis is associated with a lower cumulative probability of false-positive results compared with digital mammography; biennial vs annual screening was associated with larger reductions in cumulative false-positive risk for both modalities.

## Teaser

This comparative effectiveness study examines the probability of false-positive results associated with the use of digital breast tomosynthesis vs digital mammography.

## Introduction

Early breast cancer detection via screening mammography is a key strategy to decrease breast cancer morbidity and mortality; however, mammography results in notable harms, including false-positive results, that lead to unnecessary additional imaging and biopsy procedures, financial and opportunity costs, and patient anxiety.^1,2,3,4,5^ False-positive results are common, with 12% of patients who undergo digital screening mammograms recalled for additional workup; of those recalls, only 4.4%, or 0.53% of screening mammograms overall, result in a cancer diagnosis.^6^ A Breast Cancer Surveillance Consortium (BCSC) study including mostly film mammography estimated that after 10 years of annual screening in women aged 40 to 59 years, including their baseline mammogram, 61% of individuals would experience at least 1 false-positive recall and 7% to 9% at least 1 false-positive biopsy recommendation.^7^

Digital breast tomosynthesis is rapidly disseminating in the US, with lower recall and false-positive rates compared with screening with digital mammography.^8,9,10^ In prior BCSC research involving some of the authors of the present study, reductions in recall rates with tomosynthesis vs digital mammography were found across all Breast Imaging Reporting and Data System (BI-RADS) breast density categories except for extremely dense breasts.^11^ However, effective screening requires many examinations over multiple decades. Multimodel simulation studies^4,12,13^ and BCSC studies^7,14,15,16,17^ consistently reported that repeated biennial screening was associated with lower false-positive rates than annual screening. The goal of this study was to estimate the cumulative probabilities of at least 1 false-positive recall, short-interval follow-up recommendation, and biopsy recommendation during 10 years of annual vs biennial subsequent screening with digital breast tomosynthesis vs digital mammography by decade of age and BI-RADS breast density category using longitudinal data from the BCSC.

## Methods

### Study Setting, Data Sources, and Participants

In this observational comparative effectiveness study with prospective data collection, we selected digital mammography and digital breast tomosynthesis (ie, 3-dimensional mammography) screening examinations performed between January 1, 2005, and December 31, 2018, among women aged 40 to 79 years at 126 breast imaging facilities participating in 6 BCSC registries^18^: San Francisco Mammography Registry, New Hampshire Mammography Network, Vermont Breast Cancer Surveillance System, Carolina Mammography Registry, Metro Chicago Breast Cancer Registry, and Sacramento Area Breast Imaging Registry. Screening examinations were defined by the clinical indication. We excluded baseline examinations, unilateral examinations, examinations with a mammogram within the last 9 months, and examinations in women with a history of breast cancer or mastectomy. We restricted our focus to subsequent examinations for comparability across age groups, as baseline examinations tend to be concentrated in younger women and have higher false-positive rates than subsequent examinations.^7^ The final study cohort included 2 969 055 screening examinations among 903 495 women.

Breast Cancer Surveillance Consortium registries and the statistical coordinating center received institutional review board approval from the University of California, Davis; University of California, San Francisco; University of North Carolina at Chapel Hill; University of Illinois, Chicago; University of Vermont; Advocate Health Care; Dartmouth; and Kaiser Permanente Washington for active or passive consenting processes or a waiver of consent to enroll participants, link and pool data, and perform analysis. Procedures were Health Insurance Portability and Accountability Act compliant, and registries and the coordinating center received a Federal Certificate of Confidentiality and other protections for the identities of women, physicians, and facilities. This study followed the Strengthening the Reporting of Observational Studies in Epidemiology (STROBE) reporting guideline for cohort studies, considering the adaptations recommended by the International Society for Pharmacoeconomics and Outcomes Research (ISPOR) reporting guideline for comparative effectiveness studies.

### Measures, Definitions, and Outcomes

At each examination, self-reported information on age and time since last mammogram was obtained from questionnaires. Radiologists (N = 699) reported assessments following American College of Radiology BI-RADS terminology and recorded BI-RADS breast density as part of the clinical interpretation as almost entirely fatty, scattered fibroglandular densities, heterogeneously dense, or extremely dense.^19^ Screening interval was defined using BCSC data and self-reported information to determine the date of the previous mammogram and categorized as annual (9-18 months), biennial (19-30 months), or triennial or longer (>30 months). Screening round was based on the number of prior subsequent examinations observed for each woman.

Primary outcomes were false-positive recall, false-positive short-interval follow-up recommendation, and false-positive biopsy recommendation. Recall was defined as a BI-RADS initial assessment of 0 (needs additional imaging evaluation), 3 (probably benign finding), 4 (suspicious abnormality), or 5 (highly suggestive of cancer). Short-interval follow-up recommendation was defined as a BI-RADS final assessment of 3 after diagnostic imaging workup within 90 days of a recalled screening examination. Biopsy recommendation was defined as a BI-RADS final assessment of 4 or 5. Recalls, short-interval follow-up recommendations, and biopsy recommendations were considered false-positive if no diagnosis of invasive carcinoma or ductal carcinoma in situ occurred within 1 year of the screening examination and before the next screening examination. We imputed false-positive short-interval follow-up and biopsy recommendations for examinations with a false-positive recall but unresolved final assessment (n = 14 171 [0.5%]) based on age, breast density, screening modality, and screening interval, imputing a single value because less than 1% of data was missing.^20^ Diagnoses of invasive breast carcinoma and ductal carcinoma in situ were obtained by linkage to pathology databases and state or regional tumor registries. Death information was obtained by linkage with state death records.

### Statistical Analysis

Data analysis was performed from February 9 to September 7, 2021. We summarized cohort characteristics by screening modality. We estimated unadjusted percentages of examinations with false-positive results by age group, breast density, screening interval, and modality. Cumulative probabilities of at least 1 false-positive result after 10 years of annual or biennial screening were estimated using a discrete-time survival model to account for censoring.^21,22,23^ Logistic regression was used to estimate the probability of each outcome after a single screening mammogram as a function of age (linear and quadratic), breast density, screening interval, modality, and all interactions between these variables; screening round and its interaction with modality; and censoring round, defined as the total number of screening examinations observed for a woman.^21,22,23^ We excluded screening mammograms after the first false-positive result being modeled to estimate the probability of a first false-positive result given no earlier false-positive results. Standardized 10-year cumulative probabilities of at least 1 false-positive result after 10 annual and 5 biennial screening rounds were estimated from round-specific probabilities by marginalizing over the distribution of covariate combinations and censoring round, increasing age in 1- or 2-year increments for annual or biennial examinations, increasing screening round in 1-unit increments, and holding screening interval, screening modality, density, and censoring round constant.^21,22,23^ Cumulative probabilities were adjusted for competing risks of breast cancer diagnoses or death from any cause within 1 year of an annual or 2 years of a biennial screening mammogram and estimated with a logistic regression model including age, breast density, screening interval, modality, and all interactions.^23^ In addition, 95% CIs were estimated using a nonparametric bootstrap with 10 000 iterations.

Statistical analyses were performed using SAS/STAT software, version 14.2 (SAS Institute Inc), R, version 4.0.2 (R Foundation for Statistical Computing), and RStudio, version 1.3.1056 (RStudio Inc). Tests of statistical significance used a 2-sided α = .05.

## Results

Our study included 444 704 digital breast tomosynthesis and 2 524 351 digital mammography examinations among 903 495 women; mean (SD) age at the time of screening was 57.6 (9.9) years; 58% of the examinations were in individuals younger than 60 years, and 46% were performed in women with dense breasts (Table 1). Women underwent a mean (SD) of 3.3 (2.5) examinations. A total of 2 132 274 examinations (71.8%) were annual screening mammograms, 497 829 (16.8%) were biennial screening mammograms, and 338 952 (11.4%) were triennial or longer screening mammograms. Tomosynthesis tended to be used at later screening rounds than digital mammography, with 148 728 tomosynthesis examinations (33.4%) vs 150 606 digital examinations (6.0%) performed in round 7 or later. Overall, 33 760 tomosynthesis (7.6%) and 227 485 digital mammograms (9.0%) resulted in a false-positive recall, 7865 tomosynthesis (1.8%) and 52 236 digital mammograms (2.1%) resulted in a false-positive short-interval follow-up recommendation, and 4893 tomosynthesis (1.1%) and 30 058 digital mammograms (1.2%) resulted in a false-positive biopsy recommendation (Table 1). The distribution of age group and breast density by screening interval and modality are provided in eTable 1 in the Supplement.

**Table 1.** Characteristics of 2 969 055 Screening Mammograms Included in the Study Population of 903 495 Women

Covariate	No. (%)	
	Digital breast tomosynthesis	Digital mammography
All screening mammograms	444 704 (15.0)	2 524 351 (85.0)
Age group, y		
40-49	99 686 (22.4)	626 257 (24.8)
50-59	150 318 (33.8)	848 635 (33.6)
60-69	131 225 (29.5)	690 124 (27.3)
70-79	63 475 (14.3)	359 335 (14.2)
BI-RADS breast density		
Almost entirely fatty	41 707 (9.4)	259 241 (10.3)
Scattered fibroglandular densities	201 176 (45.2)	1 108 836 (43.9)
Heterogeneously dense	170 416 (38.3)	968 681 (38.4)
Extremely dense	31 405 (7.1)	187 593 (7.4)
Screening interval		
Annual (9-18 mo)	325 619 (73.2)	1 806 655 (71.6)
Biennial (19-30 mo)	67 834 (15.3)	429 995 (17.0)
Triennial or longer (>30 mo)	51 251 (11.5)	287 701 (11.4)
False-positive recall		
No	410 944 (92.4)	2 296 866 (91.0)
Yes	33 760 (7.6)	227 485 (9.0)
False-positive short-interval follow-up recommendation		
No	436 839 (98.2)	2 472 115 (97.9)
Yes	7865 (1.8)	52 236 (2.1)
False-positive biopsy recommendation		
No	439 811 (98.9)	2 494 293 (98.8)
Yes	4893 (1.1)	30 058 (1.2)
Screening round (after baseline screening examination)		
1st	51 440 (11.6)	715 632 (28.3)
2nd	54 803 (12.3)	611 479 (24.2)
3rd	48 865 (11.0)	424 847 (16.8)
4th	46 299 (10.4)	292 939 (11.6)
5th	47 890 (10.8)	198 149 (7.8)
6th	46 679 (10.5)	130 699 (5.2)
≥7th	148 728 (33.4)	150 606 (6.0)

Abbreviation: BI-RADS, Breast Imaging Reporting and Data System.

### False-Positive Recall

The unadjusted percentage of mammograms with a false-positive recall on tomosynthesis vs digital mammography were 6.8% (95% CI, 6.7%-6.9%) vs 8.2% (95% CI, 8.2%-8.3%; difference, –1.4; 95% CI, –1.5 to –1.3) for annual examinations and 8.5% (95% CI, 8.3%-8.7%) vs 9.5% (95% CI, 9.4%-9.6%; difference, –1.0; 95% CI, –1.2 to –0.7) for biennial examinations and were generally lower for women with almost entirely fatty breasts and for older vs younger women (eTable 2 in the Supplement). Comparing modalities, the overall cumulative probability of at least 1 false-positive recall after 10 years of screening with tomosynthesis vs digital mammography was 49.6% (95% CI, 49.0%-50.2%) vs 56.3% (95% CI, 56.0%-56.7%) for annual screening (difference, –6.7; 95% CI, –7.4 to –6.1) and 35.7% (95% CI, 34.8%-36.6%) vs 38.1% (95% CI, 37.8%-38.5%) for biennial screening (difference, –2.4; 95% CI, –3.4 to –1.5) (Table 2). Comparing biennial vs annual screening, the overall cumulative probability was lower with tomosynthesis (difference, –13.9; 95% CI, –14.9 to –12.8) and digital mammography (difference, –18.2; 95% CI, –18.6 to –17.7). Cumulative false-positive recall probabilities generally declined with increasing age (eg, 39.8 to 47.0 vs 60.8 to 68.0 among annual screening in women ages 70-79 vs 40-49 years) and decreasing breast density. For example, among women aged 40 to 49 years, the estimated probabilities of a false-positive recall after 10 years of annual screening with tomosynthesis were 31.0% (95% CI, 25.6%-36.7%) for women with entirely fatty breasts and 67.3% (95% CI, 63.8%-70.7%) for women with extremely dense breasts. Decreases in the cumulative false-positive recall probability with tomosynthesis compared with digital mammography were largest for annual screening in women with nondense breasts (differences −6.5 to −12.8). Cumulative false-positive recall probabilities among women with extremely dense breasts tended to be higher, but not significantly different, for tomosynthesis vs digital mammography regardless of screening interval (eg, annual screening age 40-49 years: 67.3%; 95% CI, 63.8%-70.7% vs 65.0%; 95% CI, 63.6%-66.4%; difference, 2.3; 95% CI, –1.4 to 6.0; biennial screening age 40-49 years: 51.2%; 95% CI, 45.7%-56.9% vs 46.1%; 95% CI, 44.1%-48.0%; difference, 5.2; 95% CI, –0.6 to 11.1).

**Table 2.** Cumulative Probability of at Least 1 False-Positive Recall After 10 Years of Screening

Age group and breast density	Cumulative probability (95% CI)							
	Annual screening			Biennial screening			Differences for biennial vs annual screening by modality	
	Digital breast tomosynthesis	Digital mammography	Difference	Digital breast tomosynthesis	Digital mammography	Difference	Digital breast tomosynthesis	Digital mammography^a^
All screening mammograms	49.6 (49.0 to 50.2)	56.3 (56.0 to 56.7)	–6.7 (–7.4 to –6.1)^a^	35.7 (34.8 to 36.6)	38.1 (37.8 to 38.5)	–2.4 (–3.4 to –1.5)^a^	–13.9 (–14.9 to –12.8)^a^	–18.2 (–18.6 to –17.7)
Age, 40-49 y	60.8 (59.4 to 62.1)	68.0 (67.4 to 68.6)	–7.2 (–8.7 to –5.8)^a^	46.1 (44.1 to 48.2)	48.7 (47.9 to 49.4)	–2.5 (–4.7 to –0.3)^a^	–14.6 (–17.1 to –12.2)^a^	–19.4 (–20.3 to –18.4)
Almost entirely fatty	31.0 (25.6 to 36.7)	39.7 (37.5 to 41.9)	–8.7 (–14.6 to –2.6)^a^	26.4 (18.5 to 34.8)	24.5 (21.9 to 27.3)	1.9 (–6.6 to 10.6)	–4.6 (–14.3 to 5.4)	–15.2 (–18.6 to –11.7)
Scattered fibroglandular densities	51.8 (49.5 to 54.2)	64.7 (63.7 to 65.6)	–12.8 (–15.3 to –10.3)^a^	38.1 (34.6 to 41.6)	44.6 (43.3 to 46.0)	–6.5 (–10.3 to –2.8)^a^	–13.7 (–18.0 to –9.4)^a^	–20.0 (–21.6 to –18.4)
Heterogeneously dense	68.0 (66.1 to 69.8)	74.2 (73.5 to 75.0)	–6.3 (–8.3 to –4.3)^a^	51.9 (48.9 to 54.9)	54.4 (53.3 to 55.5)	–2.5 (–5.7 to 0.7)	–16.1 (–19.6 to –12.6)^a^	–19.8 (–21.2 to –18.5)
Extremely dense	67.3 (63.8 to 70.7)	65.0 (63.6 to 66.4)	2.3 (–1.4 to 6.0)	51.2 (45.7 to 56.9)	46.1 (44.1 to 48.0)	5.2 (–0.6 to 11.1)	–16.0 (–22.6 to –9.5)^a^	–18.9 (–21.3 to –16.5)
Age, 50-59 y	51.1 (50.3 to 51.9)	57.6 (57.2 to 58.0)	–6.5 (–7.4 to –5.6)^a^	34.8 (33.7 to 35.9)	37.6 (37.2 to 38.0)	–2.8 (–3.9 to –1.6)^a^	–16.3 (–17.7 to –14.9)^a^	–20.0 (–20.6 to –19.5)
Almost entirely fatty	29.1 (26.6 to 31.7)	36.3 (35.3 to 37.2)	–7.2 (–9.8 to –4.4)^a^	18.3 (15.5 to 21.3)	22.6 (21.5 to 23.8)	–4.3 (–7.4 to –1.2)^a^	–10.8 (–14.6 to –6.9)^a^	–13.6 (–15.1 to –12.1)
Scattered fibroglandular densities	46.7 (45.4 to 47.9)	55.6 (55.1 to 56.2)	–9.0 (–10.3 to –7.6)^a^	31.6 (30.0 to 33.2)	36.7 (36.1 to 37.3)	–5.1 (–6.8 to –3.4)^a^	–15.1 (–17.1 to –13.1)^a^	–19.0 (–19.7 to –18.2)
Heterogeneously dense	59.4 (58.1 to 60.6)	64.7 (64.1 to 65.2)	–5.3 (–6.6 to –3.9)^a^	41.0 (39.1 to 42.8)	42.2 (41.6 to 42.9)	–1.3 (–3.2 to 0.7)	–18.4 (–20.7 to –16.2)^a^	–22.4 (–23.2 to –21.6)
Extremely dense	60.4 (57.4 to 63.4)	58.8 (57.6 to 59.9)	1.7 (–1.6 to 4.8)	42.2 (37.7 to 46.7)	36.9 (35.3 to 38.4)	5.3 (0.5 to 10.1)	–18.3 (–23.6 to –12.8)^a^	–21.9 (–23.8 to –20.0)
Age, 60-69 y	44.0 (43.2 to 44.9)	50.4 (50.0 to 50.8)	–6.3 (–7.3 to –5.4)^a^	29.3 (28.2 to 30.4)	31.7 (31.3 to 32.1)	–2.4 (–3.6 to –1.2)^a^	–14.7 (–16.1 to –13.4)^a^	–18.6 (–19.2 to –18.1)
Almost entirely fatty	27.6 (25.3 to 30.0)	34.1 (33.2 to 35.0)	–6.5 (–9.0 to –3.9)^a^	17.2 (14.6 to 20.1)	22.6 (21.5 to 23.6)	–5.3 (–8.2 to –2.3)^a^	–10.4 (–13.9 to –6.8)^a^	–11.5 (–12.9 to –10.1)
Scattered fibroglandular densities	42.5 (41.3 to 43.7)	50.2 (49.7 to 50.8)	–7.7 (–9.0 to –6.4)^a^	28.7 (27.2 to 30.3)	32.0 (31.4 to 32.6)	–3.3 (–4.9 to –1.6)^a^	–13.8 (–15.8 to –11.9)^a^	–18.2 (–19.0 to –17.4)
Heterogeneously dense	52.9 (51.6 to 54.3)	57.6 (57.1 to 58.2)	–4.7 (–6.2 to –3.3)^a^	34.9 (33.0 to 37.0)	35.6 (34.9 to 36.3)	–0.7 (–2.8 to 1.4)	–18.0 (–20.4 to –15.5)^a^	–22.0 (–22.9 to –21.1)
Extremely dense	49.0 (45.3 to 52.6)	50.2 (48.8 to 51.6)	–1.3 (–5.2 to 2.6)	34.8 (29.2 to 40.4)	29.3 (27.4 to 31.2)	5.5 (–0.4 to 11.4)	–14.2 (–20.8 to –7.5)^a^	–20.9 (–23.3 to –18.6)
Age, 70-79 y	39.8 (38.5 to 41.2)	47.0 (46.5 to 47.5)	–7.2 (–8.6 to –5.7)^a^	28.6 (26.6 to 30.7)	29.7 (29.0 to 30.4)	–1.1 (–3.2 to 1.1)	–11.2 (–13.6 to –8.7)^a^	–17.3 (–18.2 to –16.4)
Almost entirely fatty	26.5 (22.9 to 30.1)	33.0 (31.8 to 34.3)	–6.5 (–10.4 to –2.7)^a^	21.8 (16.7 to 26.8)	24.2 (22.4 to 25.9)	–2.4 (–7.9 to 3.0)	–4.8 (–11.0 to 1.4)	–8.8 (–11.0 to –6.7)
Scattered fibroglandular densities	39.3 (37.4 to 41.1)	48.0 (47.3 to 48.6)	–8.7 (–10.7 to –6.8)^a^	28.7 (26.0 to 31.4)	29.9 (28.9 to 30.9)	–1.2 (–4.0 to 1.7)	–10.6 (–13.8 to –7.3)^a^	–18.1 (–19.3 to –16.9)
Heterogeneously dense	48.3 (45.8 to 50.8)	53.0 (52.1 to 53.9)	–4.7 (–7.3 to –2.1)^a^	32.4 (28.4 to 36.4)	33.2 (31.8 to 34.6)	–0.7 (–5.1 to 3.5)	–15.9 (–20.5 to –11.2)^a^	–19.8 (–21.5 to –18.2)
Extremely dense	34.9 (27.1 to 42.6)	40.2 (37.2 to 43.1)	–5.3 (–13.6 to 2.8)	29.3 (18.0 to 40.5)	23.3 (19.3 to 27.4)	6.0 (–6.1 to 17.9)	–5.6 (–19.2 to 8.1)	–16.9 (–21.8 to –11.8)

^a^ Statistically significant.

### False-Positive Short-Interval Follow-up Recommendations

The unadjusted percentage of examinations with a false-positive short-interval follow-up recommendation on tomosynthesis vs digital mammography were 1.5% (95% CI, 1.4%-1.5%) vs 1.8% (95% CI, 1.8%-1.8%; difference, –0.3; 95% CI, –0.3 to –0.3) for annual examinations and 2.1% (95% CI, 2.0%-2.2%) vs 2.2% (95% CI, 2.2%-2.3%; difference, –0.2; 95% CI, –0.3 to 0.0) for biennial examinations and were generally lower for women with almost entirely fatty breasts and for older vs younger women (eTable 3 in the Supplement). Comparing modalities, the overall cumulative probability of at least 1 false-positive short-interval follow-up recommendation after 10 years of tomosynthesis vs digital mammography screening was 16.6% (95% CI, 16.1%-17.1%) vs 17.8% (95% CI, 17.4%-18.2%) for annual (difference, –1.1; 95% CI, –1.7 to –0.6) and 10.3% (95% CI, 9.8%-10.9%) vs 10.5% (95% CI, 10.2%-10.7%) for biennial screening (difference, –0.1; 95% CI, –0.7 to 0.5) (Table 3). Comparing biennial vs annual screening, the overall cumulative probability was lower with tomosynthesis (difference, –6.3; 95% CI, –7.0 to –5.6) and digital mammography (difference, –7.3; 95% CI, –7.7 to –6.9). In general, the cumulative probability of a false-positive short-interval follow-up recommendation were lower with increasing age (eg, 13.3 to 14.2 vs 20.7 to 20.9 among annual screening in women ages 70-79 vs 40-49 years) and decreasing breast density. Decreases with tomosynthesis were largest for annual screening in women with nondense breasts (differences 0.1 to −5.2).

**Table 3.** Cumulative Probability of at Least 1 False-Positive Short-Interval Follow-up Recommendation After 10 Years of Screening

Age group and breast density	Cumulative probability (95% CI)							
	Annual screening			Biennial screening			Differences for biennial vs annual screening by modality	
	Digital breast tomosynthesis	Digital mammography	Difference	Digital breast tomosynthesis	Digital mammography	Difference	Digital breast tomosynthesis	Digital mammography^a^
All screening mammograms	16.6 (16.1 to 17.1)	17.8 (17.4 to 18.2)	–1.1 (–1.7 to –0.6)^a^	10.3 (9.8 to 10.9)	10.5 (10.2 to 10.7)	–0.1 (–0.7 to 0.5)	–6.3 (–7.0 to –5.6)^a^	–7.3 (–7.7 to –6.9)
Age, 40-49 y	20.7 (19.6 to 21.8)	20.9 (20.4 to 21.5)	–0.3 (–1.4 to 0.9)	13.1 (11.9 to 14.4)	13.2 (12.8 to 13.7)	–0.1 (–1.4 to 1.3)	–7.5 (–9.2 to –5.9)^a^	–7.7 (–8.4 to –7.0)
Almost entirely fatty	6.6 (4.0 to 9.6)	11.8 (10.4 to 13.3)	–5.2 (–8.2 to –1.9)^a^	6.6 (3.0 to 10.9)	7.6 (6.1 to 9.3)	–1.0 (–5.0 to 3.5)	0.0 (–4.7 to 4.9)	–4.2 (–6.4 to –2.0)
Scattered fibroglandular densities	17.5 (15.8 to 19.2)	18.6 (17.8 to 19.4)	–1.1 (–3.0 to 0.7)	10.5 (8.6 to 12.6)	11.6 (10.8 to 12.4)	–1.1 (–3.1 to 1.1)	–7.0 (–9.6 to –4.3)^a^	–7.0 (–8.1 to –6.0)
Heterogeneously dense	23.5 (22.0 to 25.1)	23.3 (22.6 to 24.1)	0.2 (–1.5 to 1.9)	14.6 (12.7 to 16.5)	14.9 (14.2 to 15.6)	–0.4 (–2.4 to 1.7)	–8.9 (–11.4 to –6.5)^a^	–8.4 (–9.4 to –7.4)
Extremely dense	23.2 (20.5 to 26.0)	21.3 (20.2 to 22.5)	1.9 (–1.0 to 4.9)	16.5 (12.9 to 20.4)	13.0 (11.8 to 14.3)	3.5 (–0.3 to 7.5)	–6.7 (–11.4 to –2.1)^a^	–8.3 (–10.0 to –6.6)
Age, 50-59 y	17.0 (16.3 to 17.7)	18.5 (18.1 to 19.0)	–1.5 (–2.2 to –0.9)^a^	10.0 (9.4 to 10.7)	10.5 (10.2 to 10.7)	–0.4 (–1.1 to 0.3)	–7.0 (–7.9 to –6.1)^a^	–8.1 (–8.6 to –7.6)
Almost entirely fatty	8.9 (7.3 to 10.6)	11.6 (10.9 to 12.3)	–2.7 (–4.5 to –0.9)^a^	5.3 (3.7 to 7.0)	6.8 (6.1 to 7.5)	–1.5 (–3.2 to 0.3)	–3.6 (–5.9 to –1.2)^a^	–4.8 (–5.8 to –3.9)
Scattered fibroglandular densities	15.9 (15.0 to 16.8)	17.6 (17.1 to 18.1)	–1.7 (–2.7 to –0.8)^a^	9.9 (8.9 to 10.9)	10.1 (9.7 to 10.5)	–0.2 (–1.3 to 0.8)	–6.0 (–7.3 to –4.7)^a^	–7.5 (–8.1 to –6.9)
Heterogeneously dense	19.5 (18.5 to 20.6)	20.9 (20.4 to 21.5)	–1.4 (–2.5 to –0.3)^a^	11.1 (10.0 to 12.2)	11.8 (11.3 to 12.2)	–0.6 (–1.8 to 0.5)	–8.4 (–9.9 to –6.9)^a^	–9.2 (–9.9 to –8.5)
Extremely dense	19.8 (17.3 to 22.3)	19.5 (18.5 to 20.6)	0.2 (–2.4 to 3.0)	11.1 (8.3 to 14.2)	10.2 (9.1 to 11.2)	1.0 (–2.0 to 4.1)	–8.6 (–12.5 to –4.7)^a^	–9.4 (–10.8 to –7.9)
Age, 60-69 y	14.7 (14.1 to 15.3)	16.2 (15.8 to 16.6)	–1.5 (–2.2 to –0.9)^a^	8.7 (8.0 to 9.4)	8.8 (8.5 to 9.0)	–0.1 (–0.8 to 0.7)	–6.0 (–6.9 to –5.1)^a^	–7.5 (–7.9 to –7.0)
Almost entirely fatty	8.9 (7.4 to 10.5)	11.1 (10.4 to 11.8)	–2.2 (–3.9 to –0.5)^a^	5.5 (4.0 to 7.2)	6.7 (6.0 to 7.3)	–1.1 (–2.8 to 0.7)	–3.4 (–5.6 to –1.1)^a^	–4.4 (–5.3 to –3.5)
Scattered fibroglandular densities	14.9 (14.0 to 15.7)	16.1 (15.7 to 16.6)	–1.3 (–2.2 to –0.4)^a^	9.4 (8.4 to 10.4)	9.0 (8.6 to 9.4)	0.4 (–0.6 to 1.5)	–5.5 (–6.8 to –4.2)^a^	–7.1 (–7.7 to –6.6)
Heterogeneously dense	16.8 (15.8 to 17.8)	18.6 (18.1 to 19.2)	–1.8 (–2.9 to –0.7)^a^	9.2 (8.1 to 10.4)	9.4 (9.0 to 9.9)	–0.2 (–1.5 to 1.0)	–7.6 (–9.1 to –6.1)^a^	–9.2 (–9.9 to –8.5)
Extremely dense	14.7 (12.0 to 17.5)	14.9 (13.7 to 16.0)	–0.1 (–3.1 to 2.9)	6.3 (3.1 to 9.4)	7.3 (6.2 to 8.5)	–1.0 (–4.4 to 2.3)	–8.4 (–12.6 to –4.2)^a^	–7.5 (–9.1 to –5.9)
Age, 70-79 y	13.3 (12.3 to 14.3)	14.2 (13.8 to 14.6)	–0.9 (–2.0 to 0.2)	8.4 (7.2 to 9.7)	7.8 (7.4 to 8.3)	0.6 (–0.7 to 2.0)	–4.9 (–6.5 to –3.3)^a^	–6.4 (–7.0 to –5.8)
Almost entirely fatty	6.5 (4.5 to 8.8)	10.3 (9.4 to 11.2)	–3.8 (–6.0 to –1.4)^a^	7.5 (4.3 to 11.0)	7.2 (6.1 to 8.3)	0.3 (–3.0 to 4.0)	1.0 (–2.9 to 5.0)	–3.1 (–4.5 to –1.7)
Scattered fibroglandular densities	14.4 (13.0 to 15.8)	14.3 (13.8 to 14.9)	0.1 (–1.4 to 1.6)	9.0 (7.3 to 10.8)	8.2 (7.6 to 8.8)	0.8 (–1.0 to 2.7)	–5.4 (–7.6 to –3.1)^a^	–6.1 (–6.9 to –5.3)
Heterogeneously dense	15.1 (13.1 to 17.1)	16.4 (15.7 to 17.2)	–1.4 (–3.4 to 0.8)	8.3 (6.2 to 10.6)	7.7 (6.9 to 8.6)	0.6 (–1.8 to 3.0)	–6.7 (–9.7 to –3.8)^a^	–8.7 (–9.8 to –7.5)
Extremely dense	9.7 (5.2 to 14.9)	9.3 (7.4 to 11.2)	0.4 (–4.4 to 6.0)	3.6 (0.4 to 10.0)	5.0 (3.0 to 7.3)	–1.3 (–5.7 to 5.3)	–6.0 (–12.7 to 1.7)	–4.3 (–7.0 to –1.4)

^a^ Statistically significant.

### False-Positive Biopsy Recommendations

The unadjusted percentages of examinations with a false-positive biopsy recommendation on tomosynthesis vs digital mammography were 0.9% (95% CI, 0.9%-1.0%) vs 1.0% (95% CI, 1.0%-1.0%; difference, –0.1; 95% CI, –0.1 to 0.0) for annual examinations and 1.3% (95% CI, 1.2%-1.4%) vs 1.3% (95% CI, 1.3%-1.4%; difference, –0.1; 95% CI, –0.2 to 0.0) for biennial examinations and were generally lower for women with almost entirely fatty breasts and for older vs younger women (eTable 4 in the Supplement). Comparing modalities, the overall cumulative probability of at least 1 false-positive biopsy recommendation after 10 years of tomosynthesis vs digital mammography screening was 11.2% (95% CI, 10.7%-11.7%) vs 11.7% (95% CI, 11.4%-12.1%) for annual screening (difference, –0.5; 95% CI, –1.0 to –0.1) and 6.6% (95% CI, 6.2%-7.1%) vs 6.7% (95% CI, 6.5%-6.9%) for biennial screening (difference, –0.1; 95% CI, –0.5 to 0.4) (Table 4). Comparing biennial vs annual screening, the overall cumulative probability was lower with tomosynthesis (difference, –4.6; 95% CI, –5.2 to –3.9) and digital mammography (difference, –5.0; 95% CI, –5.4 to –4.7). In general, cumulative false-positive biopsy recommendation probabilities were lower with increasing age (eg, 9.1 to 9.3 vs 13.2 to 13.4 among annual screening in women ages 70-79 vs 40-49 years) and decreasing breast density. Decreases in the cumulative probability of false-positive biopsy recommendation were largest for annual screening in women with nondense breasts (differences −0.5 to −3.1).

**Table 4.** Cumulative Probability of at Least 1 False-Positive Biopsy Recommendation After 10 Years of Screening

Age group and breast density	Cumulative probability (95% CI)							
	Annual screening			Biennial screening			Differences for biennial vs annual screening by modality	
	Digital breast tomosynthesis	Digital mammography	Difference	Digital breast tomosynthesis	Digital mammography	Difference	Digital breast tomosynthesis	Digital mammography^a^
All screening mammograms	11.2 (10.7 to 11.7)	11.7 (11.4 to 12.1)	–0.5 (–1.0 to –0.1)^a^	6.6 (6.2 to 7.1)	6.7 (6.5 to 6.9)	–0.1 (–0.5 to 0.4)	–4.6 (–5.2 to –3.9)^a^	–5.0 (–5.4 to –4.7)
Age, 40-49 y	13.2 (12.3 to 14.2)	13.4 (12.9 to 14.0)	–0.2 (–1.2 to 0.8)	8.4 (7.4 to 9.4)	8.2 (7.8 to 8.6)	0.2 (–0.9 to 1.2)	–4.8 (–6.1 to –3.5)^a^	–5.2 (–5.8 to –4.6)
Almost entirely fatty	4.8 (2.5 to 7.4)	6.4 (5.3 to 7.6)	–1.6 (–4.2 to 1.2)	4.3 (1.5 to 8.1)	4.6 (3.4 to 5.9)	–0.2 (–3.4 to 3.7)	–0.4 (–4.3 to 3.9)	–1.8 (–3.5 to –0.1)
Scattered fibroglandular densities	10.2 (9.0 to 11.6)	10.7 (10.1 to 11.4)	–0.5 (–1.9 to 0.9)	6.6 (5.1 to 8.1)	6.6 (6.0 to 7.3)	–0.1 (–1.7 to 1.6)	–3.7 (–5.6 to –1.7)^a^	–4.1 (–5.0 to –3.2)
Heterogeneously dense	15.4 (14.1 to 16.8)	15.1 (14.4 to 15.8)	0.3 (–1.1 to 1.8)	9.5 (8.0 to 11.0)	8.9 (8.4 to 9.5)	0.6 (–1.0 to 2.2)	–5.9 (–7.9 to –3.9)^a^	–6.2 (–7.0 to –5.3)
Extremely dense	15.4 (13.1 to 17.8)	16.3 (15.2 to 17.4)	–0.9 (–3.3 to 1.7)	10.0 (7.3 to 12.9)	10.5 (9.4 to 11.6)	–0.5 (–3.4 to 2.6)	–5.4 (–9.0 to –1.7)^a^	–5.8 (–7.3 to –4.2)
Age, 50-59 y	11.7 (11.1 to 12.2)	12.4 (12.0 to 12.9)	–0.8 (–1.4 to –0.2)^a^	6.7 (6.1 to 7.3)	6.8 (6.5 to 7.0)	–0.1 (–0.7 to 0.6)	–5.0 (–5.8 to –4.1)^a^	–5.6 (–6.1 to –5.2)
Almost entirely fatty	4.9 (3.7 to 6.1)	8.0 (7.3 to 8.7)	–3.1 (–4.4 to –1.8)^a^	4.1 (2.8 to 5.7)	4.7 (4.1 to 5.3)	–0.5 (–2.0 to 1.1)	–0.7 (–2.6 to 1.1)	–3.3 (–4.2 to –2.5)
Scattered fibroglandular densities	10.5 (9.7 to 11.2)	11.0 (10.5 to 11.4)	–0.5 (–1.4 to 0.3)	5.1 (4.3 to 5.8)	6.1 (5.8 to 6.4)	–1.1 (–1.9 to –0.3)^a^	–5.4 (–6.5 to –4.3)^a^	–4.8 (–5.4 to –4.3)
Heterogeneously dense	13.8 (12.9 to 14.7)	14.4 (13.9 to 15.0)	–0.6 (–1.6 to 0.4)	8.3 (7.3 to 9.4)	7.6 (7.3 to 8.0)	0.7 (–0.4 to 1.8)	–5.5 (–6.9 to –4.1)^a^	–6.8 (–7.4 to –6.1)
Extremely dense	15.1 (12.8 to 17.4)	15.3 (14.2 to 16.3)	–0.2 (–2.7 to 2.4)	10.9 (8.2 to 14.0)	8.6 (7.6 to 9.5)	2.4 (–0.6 to 5.6)	–4.1 (–7.8 to –0.3)^a^	–6.7 (–8.1 to –5.3)
Age, 60-69 y	10.2 (9.7 to 10.8)	11.0 (10.6 to 11.4)	–0.8 (–1.3 to –0.2)^a^	5.5 (4.9 to 6.0)	5.8 (5.6 to 6.0)	–0.3 (–0.9 to 0.3)	–4.7 (–5.5 to –3.9)^a^	–5.2 (–5.6 to –4.8)
Almost entirely fatty	5.2 (4.1 to 6.4)	8.3 (7.6 to 8.9)	–3.1 (–4.3 to –1.7)^a^	4.5 (3.1 to 6.2)	4.8 (4.2 to 5.4)	–0.3 (–1.9 to 1.4)	–0.7 (–2.6 to 1.3)	–3.4 (–4.3 to –2.6)
Scattered fibroglandular densities	9.7 (9.0 to 10.4)	10.5 (10.1 to 10.9)	–0.8 (–1.6 to 0.0)	4.4 (3.8 to 5.1)	5.6 (5.3 to 5.9)	–1.2 (–1.9 to –0.4)^a^	–5.2 (–6.2 to –4.2)^a^	–4.9 (–5.4 to –4.3)
Heterogeneously dense	13.1 (12.2 to 14.1)	12.8 (12.3 to 13.3)	0.3 (–0.6 to 1.3)	7.3 (6.2 to 8.3)	6.5 (6.1 to 6.9)	0.8 (–0.3 to 1.9)	–5.9 (–7.3 to –4.4)^a^	–6.3 (–6.9 to –5.7)
Extremely dense	9.3 (6.6 to 11.9)	10.8 (9.7 to 12.0)	–1.6 (–4.4 to 1.3)	8.0 (4.7 to 11.5)	5.5 (4.4 to 6.6)	2.5 (–1.1 to 6.2)	–1.3 (–5.5 to 3.1)	–5.4 (–6.9 to –3.8)
Age, 70-79 y	9.1 (8.2 to 10.0)	9.3 (8.9 to 9.7)	–0.2 (–1.1 to 0.8)	5.1 (4.1 to 6.3)	5.1 (4.8 to 5.5)	0.0 (–1.2 to 1.2)	–4.0 (–5.4 to –2.6)^a^	–4.1 (–4.7 to –3.6)
Almost entirely fatty	5.8 (3.8 to 7.9)	7.0 (6.2 to 7.9)	–1.3 (–3.4 to 1.0)	5.5 (2.7 to 8.8)	4.9 (4.0 to 6.0)	0.6 (–2.4 to 4.0)	–0.3 (–3.8 to 3.6)	–2.1 (–3.4 to –0.8)
Scattered fibroglandular densities	8.1 (6.9 to 9.3)	9.4 (8.9 to 9.9)	–1.3 (–2.5 to 0.0)	4.5 (3.1 to 5.9)	5.2 (4.7 to 5.7)	–0.7 (–2.1 to 0.8)	–3.7 (–5.5 to –1.8)^a^	–4.2 (–4.9 to –3.5)
Heterogeneously dense	13.2 (11.4 to 15.2)	10.5 (9.8 to 11.3)	2.7 (0.7 to 4.7)	6.3 (4.2 to 8.6)	5.5 (4.7 to 6.2)	0.9 (–1.4 to 3.3)	–6.9 (–9.7 to –3.9)^a^	–5.1 (–6.1 to –4.1)
Extremely dense	3.7 (1.2 to 7.3)	5.7 (4.2 to 7.4)	–2.1 (–5.1 to 1.9)	4.4 (0.7 to 10.1)	2.8 (1.5 to 4.5)	1.6 (–2.5 to 7.5)	0.7 (–4.7 to 7.1)	–2.9 (–5.2 to –0.6)

^a^ Statistically significant.

## Discussion

We estimated the cumulative probabilities of at least 1 false-positive result after 10 years of annual or biennial screening with digital breast tomosynthesis vs digital mammography. We investigated 3 types of false-positive results occurring throughout the screening episode: recall for additional imaging, recommendation for short-interval follow-up, and recommendation for biopsy. The cumulative probability of false-positive result was lower for recall for tomosynthesis vs digital mammography with both annual and biennial screening, lower for short-interval follow-up recommendation with annual screening but not biennial screening, and similar for biopsy recommendation regardless of screening interval. All 3 types of false-positive results depended on age and breast density and were substantially lower for biennial vs annual mammography regardless of screening modality.

Prior research suggested that digital breast tomosynthesis is associated with reductions in false-positive recalls compared with digital mammography.^8,9,10^ We found that reductions in the percentage of individuals receiving at least 1 false-positive recall were modest after 10 years of subsequent screening with tomosynthesis, with reductions of 2.4% for biennial screening and 6.7% for annual screening. Nonetheless, this percentage equates to many thousands of individuals in absolute numbers, especially for annual screening, which is the dominant practice in the US.

The cumulative probability of receiving a false-positive recall under annual screening remained high with tomosynthesis, with almost half of women projected to experience at least 1 false-positive recall after 10 subsequent screens. Cumulative false-positive probabilities would be even higher if baseline examinations were considered, at which one-fifth of women are recalled for additional workup.^11^ As in earlier BCSC studies that included both film and digital mammography,^7,14,15,16,17^ we report substantial reductions in cumulative false-positive probabilities with biennial compared with annual screening, with 36% to 38% of women expected to experience at least 1 false-positive recall after 10 years of subsequent biennial screening. This reduction occurs because women undergo half as many screening examinations with biennial screening, although false-positive recalls are more common on biennial vs annual examinations. We also found large reductions with increasing age and decreasing breast density. Thus, screening interval, age, and breast density were associated with larger reductions in the cumulative probability of a false-positive recall after repeat screening than screening modality.

To our knowledge, this study is the first to estimate the cumulative probability of a false-positive short-interval follow-up recommendation after 10 years of subsequent screening, with approximately 17% of women expected to experience at least 1 short-interval follow-up recommendation under annual screening compared with 10% under biennial screening. These probabilities were only slightly lower with tomosynthesis than digital mammography. We also found the cumulative probability of at least 1 false-positive biopsy recommendation was generally similar for tomosynthesis vs digital mammography, with 1 in 9 women projected to receive a benign biopsy result after 10 years of annual screening regardless of modality. Our results showed large reductions in the cumulative false-positive biopsy recommendation probability with biennial vs annual screening and large increases with increasing breast density, similar to those reported by Kerlikowske et al^16^ in a BCSC study of mostly film mammography.

In general, women with almost entirely fatty breasts had the lowest false-positive probabilities regardless of false-positive type and screening modality and the largest reductions in cumulative false-positive probabilities with digital breast tomosynthesis vs digital mammography. In contrast, women with extremely dense breasts tended to have the highest probability of all 3 types of false-positive results and these probabilities were not significantly lower with tomosynthesis compared with digital mammography. This lack of difference in cumulative probabilities of false-positives by modality may be due to the lack of interspersed fat within dense fibroglandular tissue, with the contrast between the fat and tissue being a requirement for more accurate detection of suspicious features by interpreting radiologists. Our results are consistent with a prior BCSC study, which found that, on subsequent screening mammograms, women with extremely dense breasts did not benefit from improved recall or cancer detection with tomosynthesis.^11^ A US multi-institutional study reported improved recall rates in women with extremely dense breasts; however, the study included baseline mammograms and did not account for screening round.^24^

Previous research suggests that women receiving a false-positive recommendation for additional imaging or biopsy may experience elevated anxiety and distress, even if transient.^1,2,25,26^ We consider false-positive short-interval follow-up as a potential harm of screening because these assessments require women to return for additional diagnostic imaging 6 months after the screening mammogram, delaying receipt of the final result and potentially resulting in additional radiation exposure and pain from mammography, psychological effects, financial strain from copays and other out-of-pocket medical costs and lost work time, and opportunity costs.^27,28^ However, short-interval follow-up assessments may prevent unnecessary biopsy that poses additional harms from infection, pain, and scarring.^29^ The relative frequency and severity of these 3 types of false-positive results should be considered in evaluating the harms of screening mammograms.

Our study offers new information about the potential harms of repeated screening, which may be used to inform screening guidelines and decision-making between individuals and their physicians; however, it is important to weigh these and other potential harms with potential benefits of earlier diagnosis. Prior research shows the benefits of annual and biennial screening are similar for most women^4,7,30,31^; however, women at high risk of an advanced cancer under biennial screening, including some women with dense breasts, may reduce their risk with annual screening.^4,12,14,16,30^ Other research has found that any improvements in cancer detection are small for tomosynthesis vs digital mammography.^11,32,33,34^ Some biennial screening programs have reported larger improvements in cancer detection rates^34^; however, long-term follow-up data are needed to assess changes for nonbaseline examinations.^35^

### Limitations

This study has limitations. We excluded baseline mammograms from our analysis so that we could compare results across all age groups. Including baseline mammograms would have increased the cumulative probabilities because false-positive results are more common on baseline mammograms.^7^ We did not estimate lifetime cumulative probabilities because doing so would require extrapolation beyond the length of observation. Even with the large BCSC cohort, sample sizes were small for tomosynthesis for women with fatty and extremely dense breasts. Tomosynthesis tended to be used at later screening rounds than digital mammography because tomosynthesis diffused into clinical practice during the study period. Given the probability of false-positive results tending to decrease with screening round, adjusting for screening round as a confounder may be important for other studies, as we did in this study. Our estimates did not take into account any potential changes by calendar year, although we expect changes in false-positive rates with the diffusion of tomosynthesis to be minimal given that prior research found that early reductions in recall rates after tomosynthesis adoption were sustained.^36^ False-positive rates likely vary across facilities. Our estimates reflect the population average across 126 diverse BCSC facilities.

## Conclusions

This study noted somewhat lower cumulative probabilities of false-positive recall for digital breast tomosynthesis vs digital mammography after 10 years of annual screening, with smaller differences among women who underwent biennial screening. We did not observe consistent clinically meaningful differences in the cumulative probabilities of false-positive short-interval follow-up or biopsy recommendation by screening modality. Biennial screening interval, older age, and nondense breasts were associated with larger reductions in false-positive results than screening modality.

## Notes

Supplement. **eTable 1.** Distribution of Age Group and BI-RADS Breast Density by Screening Interval and Modality **eTable 2.** Unadjusted Probability (95% Confidence Interval) of a False-Positive Recall by Screening Interval, Modality, Age Group, and Breast Density **eTable 3.** Unadjusted Probability (95% Confidence Interval) of a False-Positive Short-Interval Follow-up Recommendation by Screening Interval, Modality, Age Group, and Breast Density **eTable 4.** Unadjusted Probability (95% Confidence Interval) of a False-Positive Biopsy Recommendation by Screening Interval, Modality, Age Group, and Breast Density
