# Supplementary material for: Cumulative Probability of False-Positive Results After 10 Years of Screening With Digital Breast Tomosynthesis vs Digital Mammography
Source: JAMA Netw Open. 2022 Mar 25;5(3):e222440. doi: 10.1001/jamanetworkopen.2022.2440 (PMC8956976; doi:10.1001/jamanetworkopen.2022.2440)
Supplement: Supplement. — eTable 1. Distribution of Age Group and BI-RADS Breast Density by Screening Interval and Modality eTable 2. Unadjusted Probability (95% Confidence Interval) of a False-Positive Recall by Screening Interval, Modality, Age Group, and Breast Density eTable 3. Unadjusted Probability (95% Confidence Interval) of a False-Positive Short-Interval Follow-up Recommendation by Screening Interval, Modality, Age Group, and Breast Density eTable 4. Unadjusted Probability (95% Confidence Interval) of a False-Positive Biopsy Recommendation by Screening Interval, Modality, Age Group, and Breast Density [file jamanetwopen-e222440-s001.pdf]

Supplementary Online Content

Ho TQH, Bissell MCS, Kerlikowske K, et al. Cumulative probability of false-positive results after 10 years of screening with digital breast tomosynthesis vs digital mammography. *JAMA Netw Open*. 2022;5(3):e222440. doi:10.1001/jamanetworkopen.2022.2440

eTable 1. Distribution of Age Group and BI-RADS Breast Density by Screening Interval and Modality

eTable 2. Unadjusted Probability (95% Confidence Interval) of a False-Positive Recall by Screening Interval, Modality, Age Group, and Breast Density

eTable 3. Unadjusted Probability (95% Confidence Interval) of a False-Positive Short-Interval Follow-up Recommendation by Screening Interval, Modality, Age Group, and Breast Density

eTable 4. Unadjusted Probability (95% Confidence Interval) of a False-Positive Biopsy Recommendation by Screening Interval, Modality, Age Group, and Breast Density

This supplementary material has been provided by the authors to give readers additional information about their work.

© 2022 Ho TQH et al. *JAMA Network Open.*

eTable 1: Distribution of age group and BI-RADS breast density by screening interval and modality.				
**Age group and breast density**	**Digital Breast Tomosynthesis**		**Digital Mammography**	
	**N**	**%**	**N**	**%**
Annual screening mammograms	325,619		1,806,655	
Age 40-49 years				
Almost Entirely Fatty	3,201	4.6%	21,904	5.2%
Scattered Fibroglandular Densities	22,331	32.0%	132,781	31.6%
Heterogeneously Dense	34,504	49.4%	205,273	48.8%
Extremely Dense	9,849	14.1%	60,270	14.3%
Age 50-59 years				
Almost Entirely Fatty	8,500	8.0%	55,042	9.3%
Scattered Fibroglandular Densities	46,083	43.1%	252,608	42.6%
Heterogeneously Dense	44,247	41.4%	238,911	40.3%
Extremely Dense	8,021	7.5%	45,865	7.7%
Age 60-69 years				
Almost Entirely Fatty	11,509	11.7%	67,495	13.2%
Scattered Fibroglandular Densities	50,555	51.4%	258,369	50.6%
Heterogeneously Dense	32,435	33.0%	165,198	32.3%
Extremely Dense	3,858	3.9%	20,027	3.9%
Age 70-79 years				
Almost Entirely Fatty	6,578	13.0%	40,737	14.4%
Scattered Fibroglandular Densities	27,671	54.8%	153,620	54.3%
Heterogeneously Dense	14,759	29.2%	81,001	28.6%
Extremely Dense	1,518	3.0%	7,554	2.7%
Biennial screening mammograms	67,834		429,995	
Age 40-49 years				
Almost Entirely Fatty	800	4.9%	5,816	5.1%
Scattered Fibroglandular Densities	5,292	32.3%	36,107	31.4%
Heterogeneously Dense	8,068	49.3%	57,458	49.9%
Extremely Dense	2,203	13.5%	15,784	13.7%
Age 50-59 years				
Almost Entirely Fatty	2,085	8.6%	14,269	9.3%
Scattered Fibroglandular Densities	10,895	45.1%	66,740	43.5%
Heterogeneously Dense	9,564	39.6%	61,581	40.1%
Extremely Dense	1,600	6.6%	10,918	7.1%
Age 60-69 years				
Almost Entirely Fatty	2,417	12.5%	15,310	13.6%
Scattered Fibroglandular Densities	10,175	52.7%	57,146	50.6%
Heterogeneously Dense	6,024	31.2%	36,307	32.1%
Extremely Dense	697	3.6%	4,220	3.7%
Age 70-79 years				
Almost Entirely Fatty	1,075	13.4%	7,648	15.8%
Scattered Fibroglandular Densities	4,525	56.5%	25,968	53.7%
Heterogeneously Dense	2,180	27.2%	13,422	27.8%

© 2022 Ho TQH et al. *JAMA Network Open.*

Extremely Dense	234	2.9%	1,301	2.7%

Abbreviation: BI-RADS, Breast Imaging Reporting and Data System.

© 2022 Ho TQH et al. *JAMA Network Open.*

eTable 2: Unadjusted probability (95% confidence interval) of a false-positive recall by screening interval, modality, age group, and breast density.			
**Age group and breast density**	**Digital Breast Tomosynthesis Probability (95% CI)**	**Digital Mammography Probability (95% CI)**	**Difference DBT vs DM (95% CI)**
All subsequent screening mammograms	7.6 (7.5, 7.7)	9.0 (9.0, 9.0)	-1.4 (-1.5, -1.3)
Annual screening mammograms	6.8 (6.7, 6.9)	8.2 (8.2, 8.3)	-1.4 (-1.5, -1.3)
Age 40-49 years	9.6 (9.4, 9.8)	10.8 (10.8, 10.9)	-1.2 (-1.5, -1.0)
Almost Entirely Fatty	3.9 (3.3, 4.7)	5.4 (5.1, 5.7)	-1.5 (-2.2, -0.8)
Scattered Fibroglandular Densities	7.6 (7.3, 8.0)	9.9 (9.7, 10.0)	-2.2 (-2.6, -1.8)
Heterogeneously Dense	10.9 (10.6, 11.2)	12.2 (12.1, 12.4)	-1.3 (-1.7, -1.0)
Extremely Dense	11.4 (10.7, 12.0)	10.3 (10.1, 10.5)	1.1 (0.4, 1.7)
Age 50-59 years	7.0 (6.8, 7.1)	8.2 (8.1, 8.3)	-1.2 (-1.4, -1.0)
Almost Entirely Fatty	3.3 (2.9, 3.7)	4.3 (4.2, 4.5)	-1.1 (-1.5, -0.7)
Scattered Fibroglandular Densities	6.2 (6.0, 6.4)	7.7 (7.6, 7.8)	-1.5 (-1.8, -1.3)
Heterogeneously Dense	8.3 (8.1, 8.6)	9.6 (9.5, 9.7)	-1.3 (-1.5, -1.0)
Extremely Dense	8.3 (7.7, 8.9)	8.3 (8.0, 8.5)	0.0 (-0.6, 0.7)
Age 60-69 years	5.5 (5.3, 5.6)	7.0 (7.0, 7.1)	-1.6 (-1.7, -1.4)
Almost Entirely Fatty	2.9 (2.6, 3.2)	4.3 (4.2, 4.5)	-1.4 (-1.8, -1.1)
Scattered Fibroglandular Densities	5.2 (5.0, 5.4)	7.0 (6.9, 7.1)	-1.8 (-2.0, -1.6)
Heterogeneously Dense	6.8 (6.5, 7.0)	8.2 (8.1, 8.4)	-1.5 (-1.8, -1.2)
Extremely Dense	5.9 (5.2, 6.6)	6.5 (6.1, 6.8)	-0.6 (-1.4, 0.2)
Age 70-79 years	5.1 (4.9, 5.3)	6.6 (6.5, 6.7)	-1.5 (-1.7, -1.3)
Almost Entirely Fatty	2.9 (2.5, 3.3)	4.2 (4.0, 4.4)	-1.4 (-1.8, -0.9)
Scattered Fibroglandular Densities	5.0 (4.7, 5.3)	6.7 (6.6, 6.8)	-1.7 (-2.0, -1.4)
Heterogeneously Dense	6.3 (5.9, 6.7)	7.8 (7.6, 7.9)	-1.4 (-1.9, -1.0)
Extremely Dense	4.2 (3.3, 5.3)	5.1 (4.7, 5.7)	-1.0 (-2.1, 0.1)
Biennial screening mammograms	8.5 (8.3, 8.7)	9.5 (9.4, 9.6)	-1.0 (-1.2, -0.7)
Age 40-49 years	11.8 (11.3, 12.3)	12.5 (12.3, 12.7)	-0.7 (-1.3, -0.2)
Almost Entirely Fatty	5.3 (3.9, 7.0)	6.0 (5.5, 6.7)	-0.8 (-2.4, 0.9)
Scattered Fibroglandular Densities	9.3 (8.6, 10.1)	11.2 (10.9, 11.6)	-1.9 (-2.8, -1.1)
Heterogeneously Dense	13.6 (12.9, 14.3)	14.2 (13.9, 14.5)	-0.6 (-1.4, 0.2)
Extremely Dense	13.5 (12.2, 15.0)	11.7 (11.2, 12.2)	1.8 (0.3, 3.3)
Age 50-59 years	8.3 (8.0, 8.7)	9.1 (8.9, 9.2)	-0.7 (-1.1, -0.4)
Almost Entirely Fatty	4.0 (3.3, 5.0)	5.0 (4.7, 5.4)	-1.0 (-1.9, -0.1)
Scattered Fibroglandular Densities	7.4 (6.9, 7.9)	8.8 (8.6, 9.0)	-1.4 (-1.9, -0.8)
Heterogeneously Dense	10.0 (9.4, 10.6)	10.4 (10.2, 10.6)	-0.4 (-1.0, 0.3)
Extremely Dense	10.3 (8.9, 11.8)	8.9 (8.4, 9.5)	1.3 (-0.2, 2.9)
Age 60-69 years	6.9 (6.5, 7.2)	7.8 (7.6, 7.9)	-0.9 (-1.3, -0.5)
Almost Entirely Fatty	4.0 (3.3, 4.9)	5.4 (5.0, 5.8)	-1.4 (-2.2, -0.5)
Scattered Fibroglandular Densities	7.0 (6.5, 7.5)	7.9 (7.7, 8.1)	-0.9 (-1.4, -0.3)
Heterogeneously Dense	7.7 (7.1, 8.4)	8.8 (8.5, 9.0)	-1.0 (-1.8, -0.3)
Extremely Dense	6.6 (5.0, 8.7)	6.7 (6.0, 7.5)	-0.1 (-2.1, 1.9)
Age 70-79 years	6.4 (5.9, 7.0)	7.4 (7.2, 7.7)	-1.0 (-1.6, -0.4)
Almost Entirely Fatty	4.4 (3.3, 5.8)	5.8 (5.3, 6.3)	-1.4 (-2.7, -0.1)
Scattered Fibroglandular Densities	6.4 (5.7, 7.2)	7.4 (7.1, 7.8)	-1.0 (-1.8, -0.2)
Heterogeneously Dense	7.6 (6.5, 8.8)	8.5 (8.1, 9.0)	-1.0 (-2.2, 0.2)
Extremely Dense	5.6 (3.3, 9.3)	6.0 (4.8, 7.4)	-0.4 (-3.6, 2.8)
Abbreviations: CI, confidence interval; DBT, digital breast tomosynthesis; DM, digital mammography.			

© 2022 Ho TQH et al. *JAMA Network Open.*

© 2022 Ho TQH et al. *JAMA Network Open.*

**eTable 3: Unadjusted probability (95% confidence interval) of a false-positive short interval follow-up recommendation by screening interval, modality, age group, and breast density.**			
**Age group and breast density**	**Digital Breast Tomosynthesis Probability (95% CI)**	**Digital Mammogram Probability (95% CI)**	**Difference DBT vs DM (95% CI)**
All subsequent screening mammograms	1.8 (1.7, 1.8)	2.1 (2.1, 2.1)	-0.3 (-0.3, -0.3)
Annual screening mammograms	1.5 (1.4, 1.5)	1.8 (1.8, 1.8)	-0.3 (-0.3, -0.3)
Age 40-49 years	2.1 (2.0, 2.2)	2.3 (2.2, 2.3)	-0.2 (-0.3, -0.1)
Almost Entirely Fatty	0.6 (0.4, 0.9)	1.2 (1.1, 1.3)	-0.6 (-0.9, -0.3)
Scattered Fibroglandular Densities	1.7 (1.5, 1.9)	2.0 (1.9, 2.1)	-0.3 (-0.5, -0.1)
Heterogeneously Dense	2.4 (2.2, 2.6)	2.5 (2.5, 2.6)	-0.1 (-0.3, 0.0)
Extremely Dense	2.3 (2.1, 2.7)	2.4 (2.2, 2.5)	0.0 (-0.3, 0.3)
Age 50-59 years	1.5 (1.5, 1.6)	1.8 (1.8, 1.8)	-0.3 (-0.3, -0.2)
Almost Entirely Fatty	0.7 (0.5, 0.9)	1.0 (0.9, 1.1)	-0.3 (-0.5, -0.1)
Scattered Fibroglandular Densities	1.4 (1.3, 1.5)	1.7 (1.7, 1.8)	-0.3 (-0.4, -0.2)
Heterogeneously Dense	1.8 (1.7, 1.9)	2.1 (2.0, 2.1)	-0.3 (-0.4, -0.1)
Extremely Dense	1.9 (1.6, 2.2)	1.9 (1.8, 2.0)	0.0 (-0.3, 0.3)
Age 60-69 years	1.2 (1.1, 1.3)	1.6 (1.5, 1.6)	-0.4 (-0.4, -0.3)
Almost Entirely Fatty	0.7 (0.6, 0.9)	1.0 (1.0, 1.1)	-0.3 (-0.5, -0.2)
Scattered Fibroglandular Densities	1.2 (1.1, 1.3)	1.6 (1.5, 1.6)	-0.3 (-0.4, -0.2)
Heterogeneously Dense	1.4 (1.2, 1.5)	1.8 (1.7, 1.9)	-0.4 (-0.6, -0.3)
Extremely Dense	1.1 (0.8, 1.4)	1.2 (1.1, 1.4)	-0.2 (-0.5, 0.2)
Age 70-79 years	1.2 (1.1, 1.3)	1.5 (1.4, 1.5)	-0.3 (-0.4, -0.2)
Almost Entirely Fatty	0.6 (0.4, 0.8)	1.0 (0.9, 1.1)	-0.4 (-0.6, -0.2)
Scattered Fibroglandular Densities	1.2 (1.1, 1.4)	1.5 (1.4, 1.5)	-0.3 (-0.4, -0.1)
Heterogeneously Dense	1.3 (1.2, 1.5)	1.8 (1.7, 1.9)	-0.4 (-0.6, -0.2)
Extremely Dense	0.9 (0.5, 1.5)	1.0 (0.8, 1.3)	-0.2 (-0.7, 0.3)
Biennial screening mammograms	2.1 (2.0, 2.2)	2.2 (2.2, 2.3)	-0.2 (-0.3, 0.0)
Age 40-49 years	2.8 (2.6, 3.1)	2.9 (2.8, 3.0)	-0.1 (-0.3, 0.2)
Almost Entirely Fatty	1.3 (0.7, 2.3)	1.7 (1.4, 2.0)	-0.4 (-1.3, 0.4)
Scattered Fibroglandular Densities	2.4 (2.0, 2.8)	2.5 (2.3, 2.6)	-0.1 (-0.6, 0.3)
Heterogeneously Dense	3.1 (2.7, 3.5)	3.3 (3.1, 3.4)	-0.2 (-0.6, 0.2)
Extremely Dense	3.5 (2.8, 4.4)	2.8 (2.5, 3.0)	0.8 (0.0, 1.6)
Age 50-59 years	1.9 (1.7, 2.1)	2.2 (2.1, 2.2)	-0.3 (-0.5, -0.1)
Almost Entirely Fatty	1.0 (0.6, 1.5)	1.3 (1.1, 1.5)	-0.3 (-0.8, 0.1)
Scattered Fibroglandular Densities	1.8 (1.5, 2.0)	2.1 (2.0, 2.3)	-0.4 (-0.6, -0.1)
Heterogeneously Dense	2.1 (1.9, 2.4)	2.4 (2.3, 2.5)	-0.3 (-0.6, 0.0)
Extremely Dense	2.4 (1.7, 3.2)	2.1 (1.9, 2.4)	0.3 (-0.5, 1.0)
Age 60-69 years	1.8 (1.7, 2.0)	1.7 (1.7, 1.8)	0.1 (-0.1, 0.3)
Almost Entirely Fatty	1.1 (0.7, 1.6)	1.4 (1.2, 1.6)	-0.3 (-0.7, 0.2)
Scattered Fibroglandular Densities	2.0 (1.8, 2.3)	1.8 (1.7, 1.9)	0.2 (0.0, 0.5)
Heterogeneously Dense	1.9 (1.6, 2.3)	1.9 (1.7, 2.0)	0.1 (-0.3, 0.5)
Extremely Dense	0.9 (0.4, 1.9)	1.4 (1.1, 1.8)	-0.6 (-1.3, 0.2)
Age 70-79 years	1.5 (1.3, 1.8)	1.8 (1.7, 1.9)	-0.3 (-0.6, 0.0)
Almost Entirely Fatty	1.4 (0.8, 2.3)	1.5 (1.3, 1.8)	-0.1 (-0.9, 0.6)
Scattered Fibroglandular Densities	1.5 (1.2, 1.9)	1.9 (1.7, 2.1)	-0.4 (-0.8, 0.0)
Heterogeneously Dense	1.7 (1.2, 2.3)	1.9 (1.7, 2.1)	-0.2 (-0.8, 0.4)

© 2022 Ho TQH et al. *JAMA Network Open.*

Extremely Dense	0.9 (0.2, 3.4)	1.4 (0.9, 2.2)	-0.5 (-1.9, 0.8)
Abbreviations: CI, confidence interval; DBT, digital breast tomosynthesis; DM, digital mammography.			

© 2022 Ho TQH et al. *JAMA Network Open.*

eTable 4: Unadjusted probability (95% confidence interval) of a false positive biopsy recommendation by screening interval, modality, age group, and breast density.

Age group and breast density	Digital Breast Tomosynthesis Probability (95% CI)	Digital Mammogram Probability (95% CI)	Difference DBT vs DM (95% CI)
All subsequent screening mammograms	1.1 (1.1, 1.1)	1.2 (1.2, 1.2)	-0.1 (-0.1, -0.1)
Annual screening mammograms	0.9 (0.9, 1.0)	1.0 (1.0, 1.0)	-0.1 (-0.1, 0.0)
Age 40-49 years	1.2 (1.2, 1.3)	1.2 (1.2, 1.2)	0.0 (-0.1, 0.1)
Almost Entirely Fatty	0.5 (0.3, 0.8)	0.6 (0.5, 0.7)	-0.1 (-0.3, 0.2)
Scattered Fibroglandular Densities	0.9 (0.8, 1.0)	1.0 (0.9, 1.0)	-0.1 (-0.2, 0.1)
Heterogeneously Dense	1.4 (1.3, 1.6)	1.3 (1.3, 1.4)	0.1 (0.0, 0.2)
Extremely Dense	1.6 (1.3, 1.8)	1.5 (1.4, 1.6)	0.1 (-0.2, 0.4)
Age 50-59 years	1.0 (0.9, 1.0)	1.0 (1.0, 1.1)	-0.1 (-0.1, 0.0)
Almost Entirely Fatty	0.3 (0.2, 0.5)	0.6 (0.5, 0.7)	-0.3 (-0.4, -0.1)
Scattered Fibroglandular Densities	0.9 (0.8, 1.0)	0.9 (0.8, 0.9)	0.0 (-0.1, 0.1)
Heterogeneously Dense	1.1 (1.0, 1.2)	1.2 (1.2, 1.3)	-0.1 (-0.2, 0.0)
Extremely Dense	1.1 (0.9, 1.3)	1.3 (1.2, 1.4)	-0.2 (-0.5, 0.0)
Age 60-69 years	0.7 (0.7, 0.8)	0.9 (0.9, 0.9)	-0.1 (-0.2, -0.1)
Almost Entirely Fatty	0.4 (0.3, 0.5)	0.7 (0.6, 0.7)	-0.3 (-0.4, -0.2)
Scattered Fibroglandular Densities	0.7 (0.6, 0.8)	0.9 (0.8, 0.9)	-0.2 (-0.3, -0.1)
Heterogeneously Dense	1.0 (0.9, 1.1)	1.0 (0.9, 1.0)	0.0 (-0.1, 0.1)
Extremely Dense	0.6 (0.4, 1.0)	0.8 (0.7, 1.0)	-0.2 (-0.5, 0.1)
Age 70-79 years	0.8 (0.7, 0.9)	0.9 (0.8, 0.9)	-0.1 (-0.2, 0.0)
Almost Entirely Fatty	0.4 (0.3, 0.6)	0.6 (0.5, 0.7)	-0.2 (-0.4, 0.0)
Scattered Fibroglandular Densities	0.7 (0.6, 0.8)	0.9 (0.8, 0.9)	-0.1 (-0.3, 0.0)
Heterogeneously Dense	1.1 (0.9, 1.3)	1.0 (0.9, 1.1)	0.1 (-0.1, 0.3)
Extremely Dense	0.4 (0.2, 0.9)	0.6 (0.5, 0.8)	-0.2 (-0.6, 0.1)
Biennial screening mammograms	1.3 (1.2, 1.4)	1.3 (1.3, 1.4)	-0.1 (-0.2, 0.0)
Age 40-49 years	1.7 (1.5, 1.9)	1.7 (1.6, 1.7)	0.1 (-0.2, 0.3)
Almost Entirely Fatty	0.8 (0.3, 1.7)	0.8 (0.6, 1.1)	-0.1 (-0.7, 0.6)
Scattered Fibroglandular Densities	1.3 (1.0, 1.6)	1.3 (1.2, 1.5)	0.0 (-0.4, 0.3)
Heterogeneously Dense	1.9 (1.7, 2.3)	1.8 (1.7, 1.9)	0.1 (-0.2, 0.5)
Extremely Dense	2.2 (1.6, 2.9)	2.1 (1.9, 2.3)	0.1 (-0.6, 0.7)
Age 50-59 years	1.3 (1.2, 1.5)	1.3 (1.2, 1.4)	0.0 (-0.1, 0.2)
Almost Entirely Fatty	0.7 (0.4, 1.2)	0.9 (0.8, 1.1)	-0.2 (-0.6, 0.2)
Scattered Fibroglandular Densities	1.0 (0.8, 1.2)	1.1 (1.0, 1.2)	-0.1 (-0.3, 0.1)
Heterogeneously Dense	1.7 (1.4, 1.9)	1.5 (1.4, 1.6)	0.2 (-0.1, 0.5)
Extremely Dense	2.1 (1.5, 2.9)	1.8 (1.5, 2.0)	0.3 (-0.5, 1.0)
Age 60-69 years	0.9 (0.8, 1.1)	1.2 (1.1, 1.3)	-0.3 (-0.4, -0.1)
Almost Entirely Fatty	0.9 (0.6, 1.4)	0.9 (0.7, 1.0)	0.0 (-0.4, 0.4)
Scattered Fibroglandular Densities	0.7 (0.6, 0.9)	1.2 (1.1, 1.3)	-0.5 (-0.7, -0.3)
Heterogeneously Dense	1.2 (0.9, 1.5)	1.3 (1.2, 1.4)	-0.1 (-0.4, 0.2)
Extremely Dense	1.4 (0.8, 2.6)	0.9 (0.7, 1.3)	0.5 (-0.4, 1.4)
Age 70-79 years	1.1 (0.9, 1.3)	1.1 (1.0, 1.2)	0.0 (-0.2, 0.2)
Almost Entirely Fatty	0.9 (0.5, 1.7)	1.1 (0.9, 1.4)	-0.2 (-0.8, 0.4)
Scattered Fibroglandular Densities	1.0 (0.7, 1.3)	1.0 (0.9, 1.2)	-0.1 (-0.4, 0.2)
Heterogeneously Dense	1.3 (0.9, 1.9)	1.1 (0.9, 1.3)	0.2 (-0.3, 0.7)

© 2022 Ho TQH et al. *JAMA Network Open.*

Extremely Dense	1.3 (0.4, 3.9)	0.6 (0.3, 1.2)	0.7 (-0.8, 2.2)
Abbreviations: CI, confidence interval; DBT, digital breast tomosynthesis; DM, digital mammography.			

© 2022 Ho TQH et al. *JAMA Network Open.*
